# Shifting microbial profiles and antimicrobial susceptibility in infectious keratitis: a 13-year longitudinal analysis at a Brazilian tertiary center

**DOI:** 10.1016/j.clinsp.2026.101220

**Published:** 2026-10-23

**Authors:** Juliana Mika Kato, Maura Salaroli Oliveira, Luiza Manhezi Shin de Oliveira, Tatiana Tanaka, Gustavo Sakuno, Luciana Santana, Evangelina da Motta Pacheco Alves de Araujo, Joyce Hisae Yamamoto, Ruth Miyuki Santo

**Affiliations:** a Department of Ophthalmology, LIM-33, Hospital das Clínicas, Faculdade de Medicina da Universidade de São Paulo (HCFMUSP), São Paulo, SP, Brazil; b Division of Infection Control, Hospital das Clínicas, Faculdade de Medicina da Universidade de São Paulo (HCFMUSP), São Paulo, SP, Brazil; c Central Laboratory Division, Hospital das Clínicas, Faculdade de Medicina da Universidade de São Paulo (HCFMUSP), São Paulo, SP, Brazil

**Keywords:** Infectious keratitis, Corneal ulcer, Antimicrobial resistance

## Abstract

**Background/Aims:**

Infectious keratitis is a sight-threatening disease often managed with empiric antibiotics. Continuous monitoring of causative agents and resistance patterns is essential to detect shifts in susceptibility and optimize treatment protocols. This study analyzed the etiology and antibiotic susceptibility trends of microbial keratitis at a major tertiary referral center over a 13-year period.

**Methods:**

Retrospective analysis of corneal scrapings from patients clinically diagnosed with infectious keratitis between January 2013 and December 2025. Cases were divided into two periods (2013–2019 and 2020–2025) to evaluate temporal trends. Statistical analyses included Bonferroni-corrected chi-square/Fisher’s exact tests and the Cochran-Armitage trend test.

**Results:**

Among 649 corneal scrapings, 343 (52.9%) were culture-positive, yielding 420 isolates (18.1% polymicrobial). Bacteria predominated (88.8%), comprising 56.9% Gram-Positive (GP) and 31.9% Gram-Negative (GN) isolates. Coagulase-negative *Staphylococci* were the most frequent GP (21.4%), while *Pseudomonas aeruginosa* (11.4%) was the leading GN. Fungi comprised for 10.2%. Longitudinal analysis revealed significant reductions in *Cutibacterium acnes* (adjusted p < 0.001) and *Serratia marcescens* (adjusted p = 0.009), a downward trend in fungal isolation (p_trend_ = 0.010) and a marginal decline in ciprofloxacin susceptibility among GN (p_trend_ = 0.056). All GP isolates demonstrated 100% *in vitro* susceptibility to vancomycin. Methicillin resistance was identified in 19.4% of *S. aureus* and 43.1% of coagulase-negative *Staphylococci*.

**Conclusion:**

The microbiological profile of infectious keratitis showed significant temporal shifts, including a reduction in *C. acnes* and a downward trend in fungal isolation. While Gram-positive and Gram-negative organisms maintained high susceptibility to vancomycin and amikacin, respectively, a marginal, non-statistically-significant decline in ciprofloxacin susceptibility among Gram-negative isolates warrants continuous microbiological surveillance.

## Highlights

• Significant rise in CNS and *S. aureus*, marked decline in *C. acnes* over 13-years.
• Ciprofloxacin susceptibility showed a downward trend among Gram-negative isolates.
• Vancomycin and amikacin maintained high *in vitro* susceptibility rates.

## Introduction

Infectious keratitis is an ophthalmologic emergency that remains a leading cause of preventable blindness worldwide. If not managed promptly, it can progress to severe complications such as corneal perforation, endophthalmitis and *phthisis bulbi*.^1^ Globally, infectious corneal ulcers represent an important public health challenge, accounting for up to 3.2% of all cases of blindness.^2^ However, the true global and economic burden is likely underestimated, particularly in developing nations where disease prevalence is higher and comprehensive surveillance remains limited.^3^

Prompt diagnosis, rapid pathogen identification, and early medical therapy are essential to prevent vision loss and long-term ocular sequelae. The current strategy of treatment relies on empirical broad-spectrum antibiotics based on clinical examination before laboratory results are available. However, clinical signs alone are frequently insufficient to distinguish between bacterial, fungal, or parasitic etiologies, even for experienced corneal specialists.^4^ As the etiology of corneal infection varies according to geography, climate, and socioeconomic factors, understanding the regional microbiological profile is crucial to characterize local epidemiology and optimize treatment protocols.^1^^,^^5^ Furthermore, the global rise of antimicrobial resistance necessitates continuous surveillance to ensure that empiric choices remain effective.

This study aimed to analyze the etiological agents and antimicrobial susceptibility patterns of isolates obtained from corneal scrapings in patients referred to a tertiary health center in Brazil over a 13-year period.

## Methods

After Institutional Ethics Committee approval, a retrospective analysis of corneal scrapings obtained from patients clinically diagnosed with infectious keratitis at a tertiary center in Brazil was conducted. This was a laboratory-based sentinel surveillance study. All microbiological examinations performed between January 2013 and December 2025 at the Institution's Central Laboratory Division were included. All procedures were performed in compliance with applicable laws and institutional guidelines, in accordance with the ethical standards of the Declaration of Helsinki.

At our Institution, corneal ulcers larger than 2 mm in diameter, centrally located, with deep stromal involvement, or unresponsive to prior treatment routinely undergo scraping for culture. Samples were collected under slit-lamp biomicroscopy visualization using topical anesthesia (0.5% proxymetacaine hydrochloride, Anestalcon™, Alcon Labs, Brazil) and sterile steel spatulas. Following sampling, empiric therapy was initiated with fortified topical antibiotics, typically comprising a cephalosporin (5% cefazolin) and an aminoglycoside (2% gentamicin) administered hourly. This empirical treatment protocol remained strictly unchanged throughout the 13-year study period.

Pathogen identification methodology evolved during the study: from 2013 to 2014, identification relied on conventional biochemical methods, whereas from 2015 onwards, Matrix-Assisted Laser Desorption Ionization Time of Flight (MALDI-TOF) mass spectrometry (Vitek MS™ system, bioMérieux, Marcy L’Etoile, France) was utilized. Antimicrobial Susceptibility Testing (AST) was performed primarily using the Vitek 2™ automated system (bioMérieux, Marcy L’Etoile, France). The disk diffusion (Kirby-Bauer) method was employed for specific organism-antibiotic combinations not available in automated panels or when confirmatory testing was required. Susceptibility results, including Zone Diameter and Minimum Inhibitory Concentration (MIC) interpretations, followed the Clinical and Laboratory Standards Institute (CLSI) M100 standards current at the time of each analysis.^6^ Original interpretations recorded in the laboratory database were maintained to reflect the contemporaneous standards of care. Moxifloxacin was included in the susceptibility testing panel for Gram-positive isolates during part of the study period but was not systematically tested across all years or organism groups; susceptibility data for this agent were therefore excluded from the formal analysis to avoid potential bias from incomplete data. For statistical analysis, intermediate susceptibility results were grouped with resistant isolates (“non-susceptible”) to provide a conservative estimate of antimicrobial resistance. Regarding specimen collection, the institutional protocol for infectious keratitis was updated in 2019 to include the use of pediatric Blood Culture Bottles (BCB). From October 2024 onwards, the protocol was streamlined to a combination of pediatric BCB, thioglycolate broth, and Brain Heart Infusion (BHI) with antibiotics.

Polymicrobial infection was defined as the isolation of two or more distinct microbial species from a single corneal scraping specimen. This definition encompassed combinations of multiple bacterial species, bacteria and fungi, or bacteria and *Acanthamoeba*. Cases yielding two or more distinct species belonging to the same genus (e.g., two distinct CNS species) were also classified as polymicrobial, as they represent co-infecting strains that may harbor clinically distinct antimicrobial resistance profiles.

The corneal scraping specimen was considered the primary unit of analysis. Repeated specimens from the same patient were included as independent specimens when obtained more than one month apart; scrapings collected within a one-month interval were considered to represent the same infectious episode and were treated as a single specimen. When multiple scrapings from the same patient occurred within this one-month window, the first specimen was retained for analysis, as it more closely reflects the pre-treatment microbiological profile of the infectious episode. Culture positivity was calculated at the specimen level, whereas organism-specific analyses were performed at the isolate level. In polymicrobial specimens, each distinct microorganism was counted separately for organism-level analyses, while each specimen contributed only once to the overall culture positivity analysis.

To evaluate temporal trends, the 13-year study period was divided into two intervals: 2013–2019 and 2020–2025. The 2020 cutoff was selected as a balanced temporal midpoint, allowing for robust statistical comparisons between two cohorts of similar duration (7-years vs. 6-years). This split also serves to investigate potential shifts in microbiological patterns and susceptibility following the onset of the COVID-19 pandemic, which impacted healthcare-seeking behavior and antibiotic stewardship globally. For comparisons between the two periods, the Chi-Square or Fisher’s exact test was used with Bonferroni correction applied separately for each group of comparisons: 23 independent comparisons for pathogen prevalence, 5 for Gram-positive antimicrobial susceptibility, and 5 for Gram-negative antimicrobial susceptibility. To complement this division and identify gradual longitudinal shifts, year-by-year analyses were conducted using Cochran-Armitage trend tests across the entire study duration. Years with missing denominators (zero isolates tested for a specific antimicrobial) were excluded from the specific trend analysis. For the trend test, a p-value < 0.05 was used to identify emerging trends, while for period comparisons, the threshold was adjusted via Bonferroni. Statistical analysis was performed using R software (version 4.2.2, R Foundation for Statistical Computing, Vienna, Austria).

## Results

Over the 13-year study period, a total of 649 corneal scrapings were performed. The cohort included 395 male (60.9%) and 254 female (39.1%), with a mean age of 52.1±19.9 years. Culture positivity was achieved in 343 samples (52.9%), yielding 420 isolated organisms. Polymicrobial infections occurred in 62 of the 343 positive samples (18.1%; 95% CI: 14.4–22.5%).

Gram-positive bacteria were the most frequent isolates (56.9%), with Coagulase-Negative *Staphylococci* (CNS) being the predominant species (21.4%). *Pseudomonas aeruginosa* was the leading Gram-negative pathogen (11.4%). Fungal keratitis accounted for 10.2% of all positive cultures. A detailed overview of the isolates is provided in Table 1.

**Table 1:** Temporal trends and comparative prevalence of microbial isolates in infectious keratitis cases by study period (2013–2019 and 2020–2025).

	2013 – 2019		2020 – 2025		Total		Adjusted p	p-trend
	N	%	N	%	N	%		
**Bacteria**	**225**	**85.2%**	**148**	**94.9%**	**373**	**88.8%**	**0.050**	**<0.001**
**Gram-positive**	**135**	**51.1%**	**104**	**66.7%**	**239**	**56.9%**	**0.051**	**<0.001**
Coagulase-negative *Staphylococci*	43	16.3%	47	30.1%	90	21.4%	0.030	<0.001
*Staphylococcus aureus*	13	4.9%	20	12.8%	33	7.9%	0.109	<0.001
*Streptococcus pneumoniae*	17	6.4%	16	10.3%	33	7.9%	>0.99	0.067
Other *Streptococcus* spp	4	1.5%	4	2.6%	8	1.9%	>0.99	0.190
*Cutibacterium acnes*	35	13.3%	3	1.9%	38	9.0%	<0.001	0.007
*Corynebacterium* sp	18	6.8%	6	3.8%	24	5.7%	>0.99	0.961
*Enterococcus faecalis*	2	0.8%	2	1.3%	4	1.0%	>0.99	0.268
Other Gram-positive bacteria	3	1.1%	6	3.8%	9	2.1%	>0.99	0.195
**Gram-negative**	**90**	**34.1%**	**44**	**28.2%**	**134**	**31.9%**	>0.99	**0.685**
*Pseudomonas aeruginosa*	29	11.0%	19	12.2%	48	11.4%	>0.99	0.851
*Serratia marcescens*	26	9.8%	2	1.3%	28	6.7%	0.009	0.108
*Klebsiella* spp	6	2.3%	1	0.6%	7	1.7%	>0.99	0.416
*Moraxella* sp	9	3.4%	10	6.4%	19	4.5%	>0.99	0.203
*Morganella morganii*	4	1.5%	3	1.9%	7	1.7%	>0.99	0.833
Other Gram-negative bacteria	16	6.1%	9	5.8%	25	6.0%	>0.99	0.840
**Fungi**	**35**	**13.3%**	**8**	**5.1%**	**43**	**10.2%**	**0.173**	**0.010**
*Fusarium* sp	18	6.8%	7	4.5%	25	6.0%	>0.99	0.575
*Candida* spp	7	2.7%	1	0.6%	8	1.9%	>0.99	0.055
*Aspergillus* sp	3	1.1%	0	0%	3	0.7%	>0.99	0.415
Other fungi	7	2.7%	0	0%	7	1.7%	>0.99	0.010
**Acanthamoeba sp**	**4**	**1.5%**	**0**	**0%**	**4**	**1.0%**	>0.99	**0.901**
**Total**	**264**	100%	**156**	**100%**	**420**	**100%**		

p for trend values were calculated using the Cochran-Armitage trend test to evaluate monotonic temporal changes across the 13-year study period and were interpreted using the conventional significance threshold of p < 0.05 (without adjustment for multiple comparisons). p-values for comparisons between study periods were calculated using the Chi-Square or Fisher's exact test and adjusted for multiple comparisons using the Bonferroni correction across 23 simultaneous comparisons; statistical significance after correction was defined as adjusted p < 0.05. Percentages were calculated relative to the total number of isolates within each study period.

In the first period (2013–2019), culture positivity was 51.4% (217/422), with CNS and *Cutibacterium acnes* identified as the most frequent isolates. In the second period (2020–2025), positivity was 55.5% (126/227), with CNS, *S. aureus* and *P. aeruginosa* predominating. Overall, bacterial isolates showed a significant longitudinal increase over the study period (p_trend_ < 0.001), driven primarily by a rise in Gram-positive organisms (p_trend_ < 0.001). Statistical analysis revealed a significant increase in the proportion of CNS isolation across both the categorical comparison (adjusted p = 0.030) and the longitudinal trend (p_trend_ < 0.001). In contrast, while the increase in *S. aureus* did not reach significance between periods (adjusted p = 0.109), a significant linear upward trend was confirmed (p_trend_ < 0.001), indicating a gradual rise over the 13-year span. Regarding declining pathogens, the reduction in *C. acnes* was statistically significant across both analytical approaches (adjusted p < 0.001; p_trend_ = 0.007). Conversely, for *Serratia marcescens,* a significant decrease was found only in the categorical comparison (adjusted p = 0.009), whereas fungal isolates showed a significant linear downward trend (p_trend_ = 0.010) despite non-significant categorical results. The detailed frequency of all isolates per year is provided in Supplementary Table 1.

Regarding the composition of the polymicrobial infections, mixed infections containing both Gram-positive and Gram-negative bacteria were the most frequent (25/62, 40.3%), followed by exclusively Gram-positive combinations (22/62, 35.5%). Fungal-bacterial associations accounted for 12.9% (8/62) of cases. CNS were the most frequent pathogens identified in polymicrobial infections, present in 47 of the 62 cases (75.8%). The leading organism combinations were CNS with *Moraxella* spp. (n = 5), followed by cases involving two distinct CNS species (n = 4) and CNS with *S. aureus* (n = 4). Detailed combinations of organisms are provided in Supplementary Table 2. The prevalence of polymicrobial keratitis remained stable between the two study periods (18.0% in 2013–2019 vs. 18.3% in 2020–2025; p > 0.99), as did the internal composition of these infections. Specifically, no significant shifts were observed in the proportions of Gram-positive and Gram-negative pairings (43.6% in 2013–2019 vs. 34.8% in 2020–2025; p = 0.596) or Gram-positive and Gram-positive combinations (28.2% in 2013–2019 vs. 47.8% in 2020–2025; p = 0.113).

For Gram-positive isolates, susceptibility rates were 100% (95% CI 97.3–100) for vancomycin, 88.7% (95% CI 80.8–93.5) for gentamicin, 84.3% (95%CI 76.2–89.9) for levofloxacin, 64.6% (95% CI 54.6–73.4) for oxacillin, and 34.7% (95% CI 26.8–43.5) for penicillin. Methicillin/oxacillin resistance was identified in 19.4% (6/31) of *S. aureus* (MRSA) and 43.1% (28/65) of CNS (MRCNS). Susceptibility rates for Gram-positive isolates remained stable for vancomycin, gentamicin, levofloxacin and oxacillin within the limitations of our sample size (Fig. 1). Longitudinal trend analysis was not performed for penicillin due to discontinuous data across the study period (data not reported in 2015, 2018, 2021, 2024, and 2025), including the two final years of the study, which would preclude a valid assessment of the complete 13-year temporal trend. Furthermore, the categorical comparison between periods showed no significant difference (adjusted p = 0.395). Annual data are detailed in Supplementary Table 3.

**Fig. 1:**
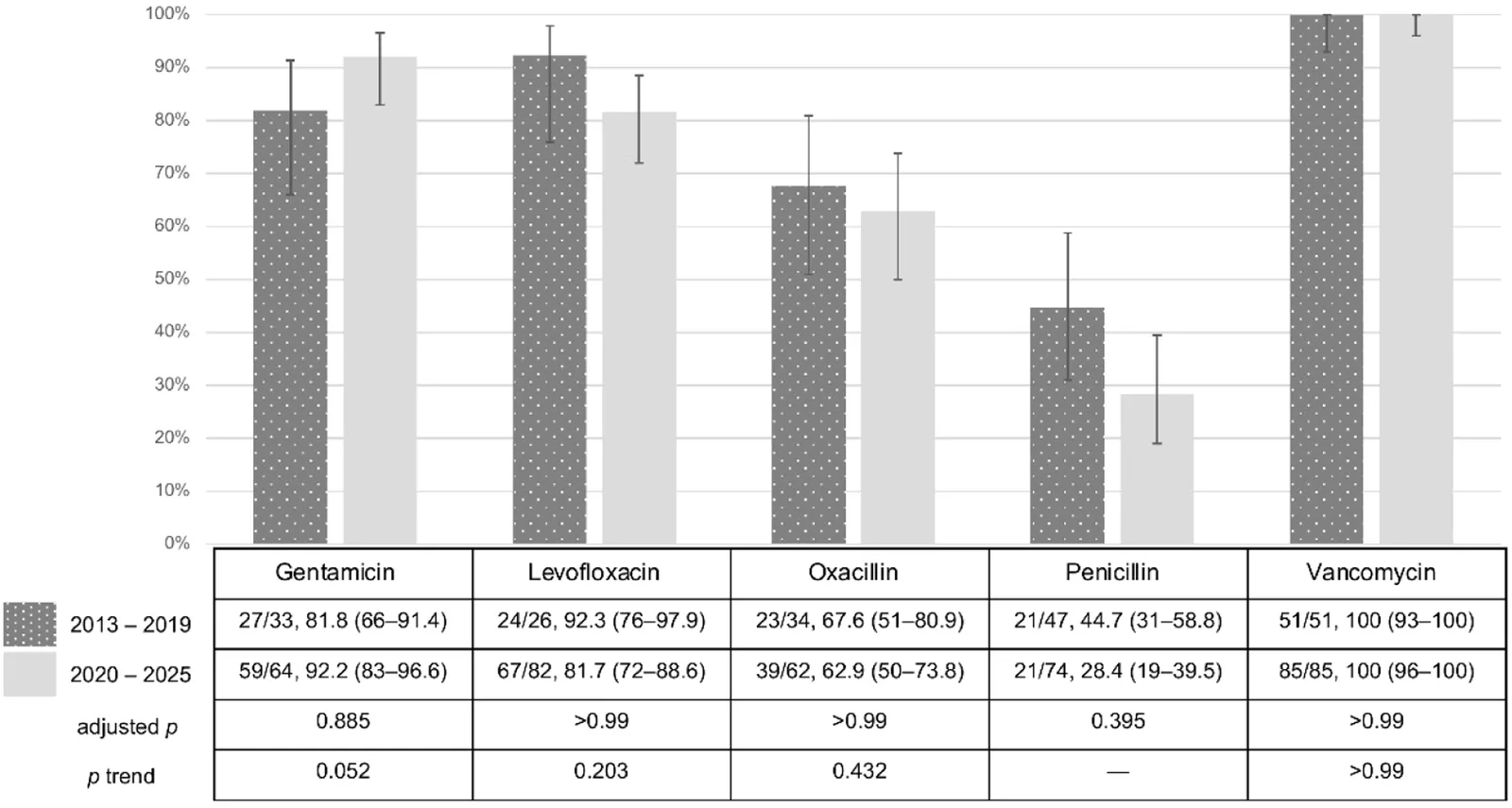
Antibiotic susceptibility of Gram-positive isolates in two study periods (2013–2019 and 2020–2025). Bars represent the percentage of susceptible isolates. The 95% Confidence Intervals (95% CI) are indicated by the error bars. The data table below the X-axis provides the raw values as follows: n/N, % (95% CI), where n is the number of susceptible isolates and N is the total number of isolates tested for each specific antibiotic. p-values for comparisons between study periods were calculated using Fisher's exact test and adjusted for multiple comparisons using Bonferroni correction across five antimicrobial agents; statistical significance after correction was defined as adjusted p < 0.05. p for trend values represents longitudinal trend analyses across the entire 13-year study period and were interpreted using a significance threshold of p < 0.05 without adjustment for multiple comparisons. Longitudinal trend analysis was not performed for Penicillin due to discontinuous data (data not reported in 2015, 2018, 2021, 2024 and 2025).

For Gram-negative isolates, susceptibility rates remained high for meropenem (100%, 95% CI 96.6–100), amikacin (99.1%, 95% CI 94.9–99.8), ceftazidime (99.1%, 95% CI 95.0–99.8), and gentamicin (97.0%, 95% CI 91.5–99.0). Regarding ciprofloxacin, a marginal downward trend in susceptibility was observed over the 13-year period (p_trend_ = 0.056), with resistance clusters emerging primarily between 2022 and 2024. Detailed annual denominators (N) and susceptibility percentages for all Gram-negative organism-antibiotic combinations are provided in Fig. 2 and Supplementary Table 3. Only one isolate of *P. aeruginosa* exhibited resistance to ceftazidime, while remaining susceptible to ciprofloxacin.

**Fig. 2:**
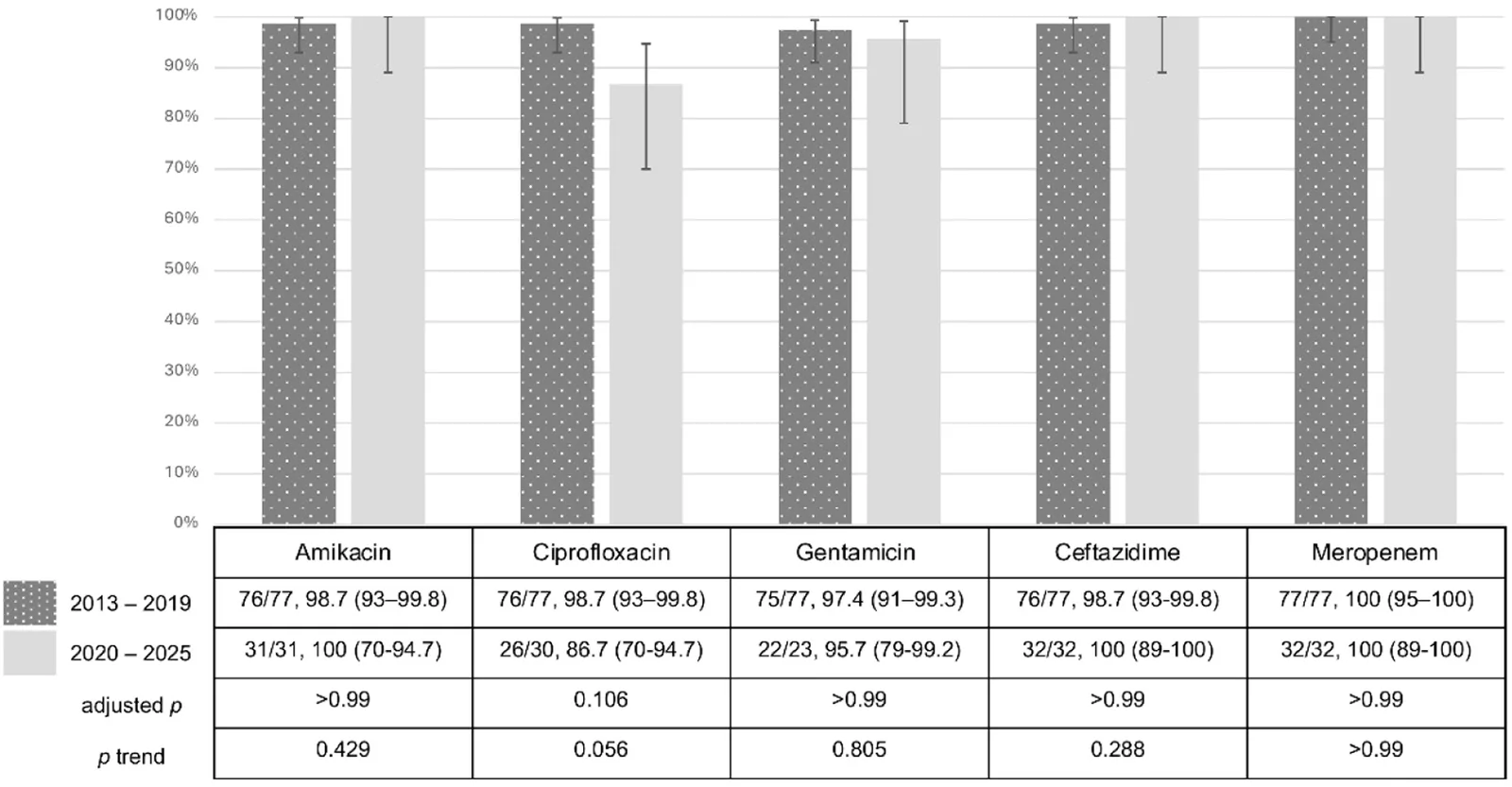
Antibiotic susceptibility of Gram-negative isolates in two study periods (2013–2019 and 2020–2025). Bars represent the percentage of susceptible isolates. The 95% Confidence Intervals (95% CI) are indicated by the error bars. The data table below the X-axis provides the raw values as follows: n/N, % (95% CI), where n is the number of susceptible isolates and N is the total number of isolates tested for each specific antibiotic. p-values for comparisons between study periods were calculated using Fisher's exact test and adjusted for multiple comparisons using Bonferroni correction across five antimicrobial agents; statistical significance after correction was defined as adjusted p < 0.05. p for trend values represents longitudinal trend analyses across the entire 13-year study period and were interpreted using a significance threshold of p < 0.05 without adjustment for multiple comparisons.

## Discussion

Infectious keratitis remains a potentially blinding condition; therefore, rapid and accurate identification of the causative organism is imperative to guide management and optimize visual outcomes. This study investigated the microbiological profile of infectious keratitis at a major tertiary referral center in Brazil ‒ the largest hospital complex in Latin America.

Over the 13-year period, our overall positivity rate was 52.9%, consistent with international benchmarks ranging from 32.6% to 78.3%.^7^, ^8^, ^9^, ^10^, ^11^ Several factors may explain the negative cultures in our series. As a high-complexity referral unit, many patients (up to 50% in previous reports from our center)^12^ were already receiving topical antibiotics prior to sampling. Additionally, the variability in corneal scraping techniques among rotating medical residents may have influenced the diagnostic yield. Procedural and technological updates also occurred throughout this extended study period, including the introduction of MALDI-TOF mass spectrometry in 2015 and modifications to the specimen collection protocol in 2019 and October 2024, incorporating pediatric BCB. Although these changes were implemented to optimize microbiological recovery and organism identification, they may also have introduced variation in long-term culture positivity and isolate recovery, which should be considered when interpreting temporal trends.

Bacterial isolates accounted for the majority of cases (88.8%), mirroring trends in North America and Europe (69.8% to 90.6%).^7^^,^^11^^,^^13^ Our findings reinforce the regional dichotomy within Brazil; while our Southeast-based center showed a predominance of Gram-positive bacteria, the warmer, more humid Northern region traditionally report a higher burden of fungal pathogens (Fig. 3).^14^, ^15^, ^16^, ^17^, ^18^, ^19^, ^20^, ^21^, ^22^ These variations are likely driven by Brazil's vast territory and diverse climatic conditions. A single study from Uberlandia, in southeast, found an unusually high proportion of fungal isolates.^17^ Nevertheless, direct comparisons among these historical cohorts should be interpreted with caution because of methodological heterogeneity. The studies were conducted over different time periods and employed diverse laboratory protocols, ranging from conventional phenotypic identification methods to MALDI-TOF mass spectrometry, which may have influenced pathogen identification and the reported microbiological profiles.

**Fig. 3:**
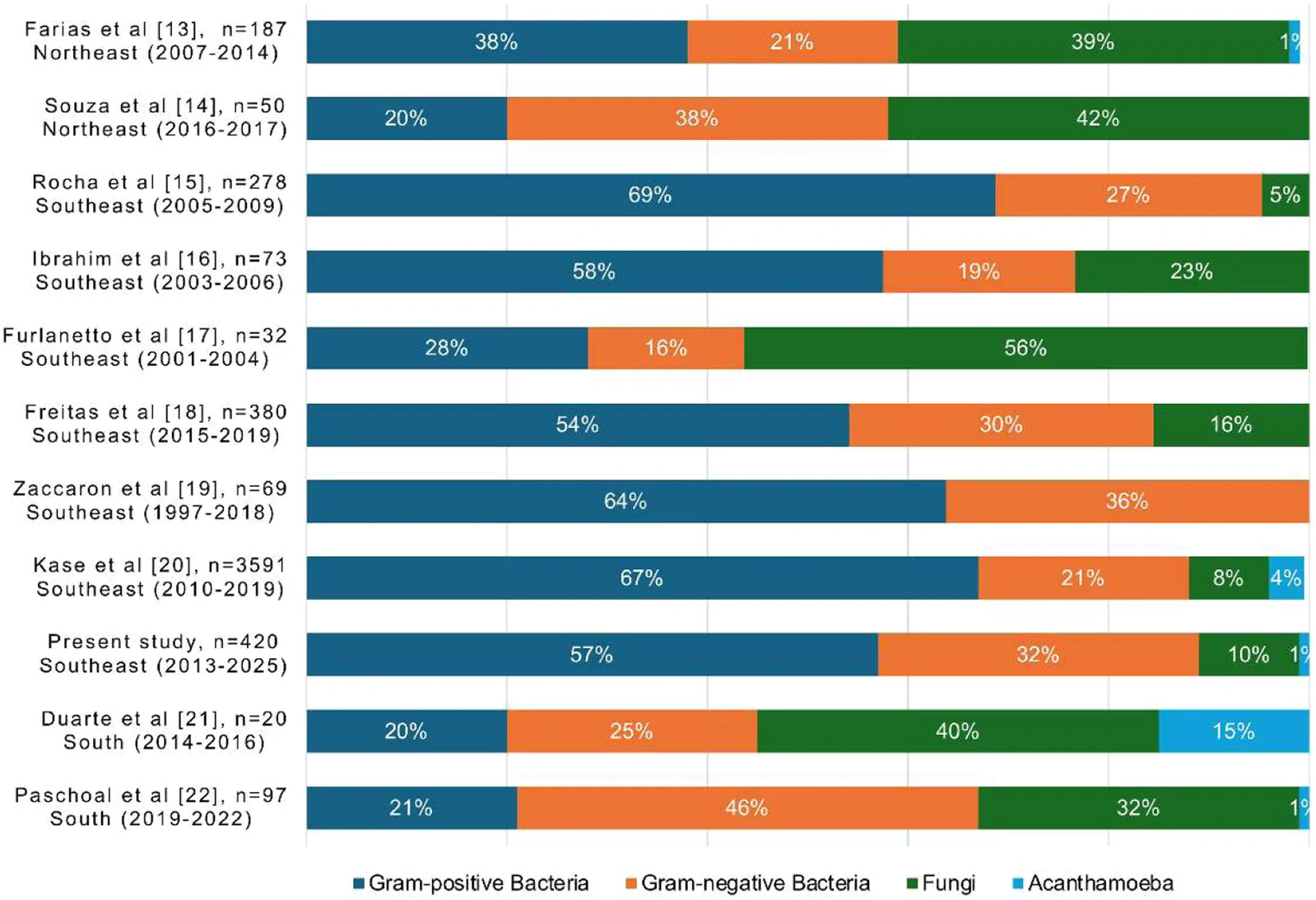
Comparative distribution of infectious keratitis pathogens across major Brazilian longitudinal studies. The chart displays the relative prevalence of Gram-positive bacteria, Gram-negative bacteria, fungi, and *Acanthamoeba*, sorted by geographic region and study period. The sample size (n) reported next to each study represents the total number of culture-positive microbial isolates in that series and serves as the denominator for all reported percentages (culture-negative cases were excluded). Diagnostic methodologies, culture protocols, and microbial identification techniques varied across institutions and study periods; therefore, direct comparisons between studies should be interpreted with caution.

A significant finding in our series was the marked decrease in *Cutibacterium acnes* identification, falling from 13.3% in the first period to 1.9% in the second period (adjusted p < 0.001, p_trend_ = 0.007), alongside a concomitant rise in the proportion of CNS (p_trend_ < 0.001) and *S. aureus* (p_trend_ < 0.001). As these organisms are common components of the eyelid microbiota,^23^ the mechanism underlying these trends cannot be definitively determined from the available retrospective data. A parsimonious explanation may involve shifts in laboratory identification methodology or subtle variations in clinical sampling techniques and incubation conditions over time. Specifically, the implementation of MALDI-TOF in 2015 and the addition of pediatric BCB to the sampling protocol in 2019 may have enhanced the rapid recovery and precise identification of *Staphylococcus* species. Given that these potential confounders were not systematically controlled, this finding should be regarded as hypothesis-generating rather than a definitive epidemiological shift.

Among Gram-negative species, *P. aeruginosa* was the predominant agent (11.4%), consistent with global rates (2.2% to 24.4%).^7^, ^8^, ^9^, ^10^, ^11^^,^^20^ A significant reduction was observed in *S. marcescens*, from 9.8% in the first period to 1.3% in the second (adjusted p = 0.009). The year-by-year analysis revealed that no *S. marcescens* isolates were recovered between 2020 and 2023, with only single isolates identified in 2024 and 2025, indicating an abrupt rather than gradual decline (Supplementary Table 1). This pattern accounts for the divergence between the significant categorical comparison (adjusted p = 0.009) and the non-significant longitudinal trend test (p_trend_ = 0.108): the Cochran-Armitage test, optimized for detecting gradual linear trends in proportions, has limited sensitivity for abrupt, concentrated shifts of this nature. The reasons for this decline remain uncertain and may reflect wider epidemiological shifts or changes in the referral patterns of patients to our tertiary center over the 13-year span.

Fungal isolates accounted for 10.2% of cases, with *Fusarium* being the most common (6.0%). Although mycotic keratitis is highly prevalent in tropical and developing regions–particularly among rural populations where agricultural trauma involving organic material is a primary risk factor,^24^^,^^25^ a nominal decline in fungal isolations was observed from 13.3% in the first period to 5.1% in the second, which did not reach statistical significance after Bonferroni correction (p = 0.173). In the absence of demographic or occupational data, the drivers behind this observation remain speculative and warrant prospective investigation. Of note, the significant trend observed for 'Other fungi' subcategory (p_trend_ = 0.010) should be interpreted with caution: this group comprises multiple rare and heterogeneous species with insufficient annual denominators for individual analysis, and the trend likely reflects the broader decline in overall fungal isolation rather than a shift in any specific pathogen.

*Acanthamoeba* keratitis cases remained rare throughout the study period, with only four cases identified, all occurring in the first study period. This apparent absence in the second period should be interpreted with caution and likely reflects diagnostic challenges rather than a true epidemiological shift. The laboratory recovery of *Acanthamoeba* requires specialized culture media, which may limit routine detection, and over the last decade, there has been an increasing clinical reliance on non-culture-based diagnostic modalities, such as in vivo confocal microscopy.^26^ Because this tools allows for rapid, independent confirmation at the bedside, many contemporary cases may be diagnosed and managed clinically without being captured in traditional microbiological culture registries. Furthermore, given the laboratory-based surveillance nature of this study, comprehensive clinical history regarding contact lens wear patterns or hygiene habits was not systematically available for the entire cohort, preventing a deeper contextual analysis of risk factors for these specific opportunistic pathogens. Nevertheless, the presence of specific risk factors and highly suggestive clinical features often justifies early empiric treatment for both fungal and protozoan keratitis even in the face of negative cultures.

The prevalence of polymicrobial keratitis remained stable throughout the study, representing a consistent challenge at our center. This temporal stability was also reflected in the composition of these mixed infections, with no significant shifts in microbiological pairings over the 13-year period. Notably, 40.3% of these cases involved mixed Gram-positive and Gram-negative combinations, highlighting the need for empiric antimicrobial coverage beyond standard Gram-positive-targeted monotherapy. Because clinical records were unavailable to evaluate patient-specific risk factors or outcomes in our cohort, we could not determine the clinical significance of these mixed infections. Nevertheless, they may pose additional therapeutic challenges because of potential synergistic interactions between pathogens. Although not reaching statistical significance (p = 0.113), a numerical increase in Gram-positive and Gram-positive combinations was observed (28.2% in 2013–2019 vs. 47.8% in 2020–2025), consistent with the overall shift toward Gram-positive predominance observed in our full cohort. This trend, however, should be interpreted with caution given the limited number of polymicrobial cases. Additionally, 12.9% involved concurrent bacterial and fungal organisms, a combination that may delay appropriate antifungal therapy when the initial culture report identifies only the bacterial component.

All Gram-positive isolates demonstrated 100% in vitro susceptibility to vancomycin, reaffirming its sustained microbiological potency against these pathogens. While rare cases of vancomycin-resistant keratitis have been previously reported in literature,^27^ our findings provide microbiological support for the continued use of fortified vancomycin within our institutional susceptibility profile. Regarding methicillin resistance, our rates (19.4% for MRSA and 43.1% for MRCNS) remained stable during the 13-year period. Our MRSA prevalence is lower than that reported in the United States by the ARMOR surveillance study, which identified methicillin resistance in 34.9% of *S. aureus* isolates.^28^

The analysis of Gram-negative isolates revealed a decrease in ciprofloxacin susceptibility, from 98.7% in 2013–2019 to 86.7% in 2020–2025. While this difference was attenuated after multiple testing correction (adjusted p = 0.275), and the longitudinal trend remained marginal (p_trend_ = 0.056), this pattern warrants continued microbiological surveillance given the critical role of ciprofloxacin in the empiric management of infectious keratitis. This observation is consistent with trends reported across Asia and other developing regions.^29^^,^^30^ For instance, despite a small sample size (n = 15), Obaid et al. documented 85% ciprofloxacin resistance rate in *P. aeruginosa* isolates from microbial keratitis.^30^ Such findings stand in contrast to previous Asian reports of 17.6% resistance^31^ and European data, where quinolones resistance generally remains below 10%.^7^^,^^32^ This escalating resistance likely reflects the widespread, and often inappropriate, empiric use of fluoroquinolones in primary care and postoperative prophylaxis, which exerts selective pressure for resistant strains. Furthermore, although our study did not specifically include fourth-generation fluoroquinolones such as moxifloxacin, international evidence indicates that ciprofloxacin resistance often serves as a precursor to reduced susceptibility across the entire class due to overlapping resistance mechanisms,^33^ reinforcing the importance of ongoing institutional surveillance. Regarding aminoglycosides, amikacin demonstrated high in vitro susceptibility (99.1%); however, its clinical efficacy may be limited by poor corneal penetration, particularly through an intact epithelium. Microbiological data should be interpreted alongside pharmacokinetic properties and clinical severity when considering therapeutic choices. Notably, the single ceftazidime-resistant *P. aeruginosa* isolate identified in our cohort remained fully susceptible to ciprofloxacin. Although based on a single isolate, this uncommon susceptibility profile illustrates that resistance patterns may not always follow expected phenotypes and reinforces the importance of culture and antimicrobial susceptibility testing to guide therapy when empirical treatment requires modification.

## Limitations

Our study has limitations inherent to its retrospective, laboratory-based sentinel surveillance design. Comprehensive clinical data, such as specific contact lens wear patterns, hygiene habits, systematic patient follow-up, and pre-referral exposure to commercial ophthalmic antibiotics (like fluoroquinolones or tobramycin), were unavailable. This precluded a more detailed assessment of risk factors for opportunistic pathogens such as *Acanthamoeba*, evaluation of clinical outcomes in polymicrobial infections, and determination of whether *in vitro* resistance translated into clinical treatment failure. Although repeated specimens from the same patient obtained more than one month apart were considered independent specimens, laterality was not systematically recorded, so we cannot exclude the possibility that some specimens represented bilateral disease rather than independent recurrent episodes. Also, the number of scrapings excluded as same-episode specimens within the 1-month window was not retrospectively quantified. Because CLSI breakpoints are updated annually, susceptibility classifications from the early study years may differ from those obtained by applying current (2023–2025) breakpoints to historical isolates. This may have introduced a source of heterogeneity into temporal analyses of antimicrobial susceptibility, particularly for antibiotic–organism combinations in which breakpoints changed substantially over the study period. In addition, antimicrobial susceptibility results may not reflect in vivo efficacy due to ocular surface pharmacodynamics, host immune response, and the high tissue concentrations achieved with topical fortified antibiotics, which can sometimes overcome laboratory-defined resistance. Some positive cultures may also represent commensal organisms from the ocular surface microbiota.

Selection bias is inherent to our institutional protocol, as corneal scrapings are only performed for severe, central, or treatment-refractory ulcers, potentially overrepresenting more virulent or resistant pathogens. However, the institutional empirical treatment protocol (5% cefazolin and 2% gentamicin) remained strictly unchanged throughout the 13-year period, which mitigates the risk of protocol-driven selection pressure as a confounder for the observed temporal trends. The retrospective design also precluded analysis of susceptibility to other commonly used agents, such as moxifloxacin, and molecular diagnostic methods (e.g., PCR or metagenomics) were not performed.

Temporal trends should be interpreted with caution. The 2020 cutoff, although useful for group comparison, may reflect changes in healthcare-seeking behavior and infection control practices during the COVID-19 pandemic rather than true microbiological shifts. In addition, unmeasured changes in patient demographics and referral patterns over the 13-year period may have influenced the findings. The introduction of MALDI-TOF in 2015 and the modifications in the specimen collection protocol in 2019 and October 2024 (the inclusion of pediatric BCB) may also have impacted organism recovery and isolation rates over time.

The study has limited statistical power to detect small differences in low-frequency pathogens. No a priori sample size calculation was performed, and non-significant results may reflect type II error. Although Bonferroni correction and 95% confidence intervals were used, the relatively small sample size remains a limitation. Specifically, the small subset of polymicrobial infections (N = 62) precluded a statistically robust comparison of resistance patterns between polymicrobial and monomicrobial isolates, as further stratification by specific bacterial species and tested antimicrobials would result in highly underpowered subgroups. Additionally, the retrospective and anonymized nature of our database prevented us from linking individual susceptibility profiles back to specific polymicrobial cultures.

Finally, these findings reflect a single-center experience. Given the geographic variability of infectious keratitis, these findings may not be generalized to other regions or primary care settings.

## Conclusion

This 13-year analysis updates the microbiological profile of infectious keratitis at a Brazilian tertiary referral center and demonstrates that the disease remains predominantly bacterial, with Gram-positive organisms as the main pathogens. A significant longitudinal increase in Gram-positive isolates was observed, driven by rising proportions of CNS and *S. aureus*. The sustained high *in vitro* susceptibility of Gram-positive and Gram-negative isolates to vancomycin and amikacin, respectively, provides microbiological support for their continued consideration in empiric protocols for severe cases, while the marginal, non-statistically-significant downward trend in ciprofloxacin susceptibility reinforces the need for ongoing microbiological surveillance. However, given the inherent limitations of this sentinel surveillance model, including the lack of clinical correlation and the specific pharmacokinetic properties of topical agents, prospective clinical trials are necessary to confirm whether these *in vitro* trends translate into clinical outcomes before formal changes to established treatment guidelines can be recommended.

## Declaration of generative AI and AI-assisted technologies in the manuscript preparation process

During the preparation of this manuscript, the authors used ChatGPT (OpenAI) to assist with language refinement. The authors reviewed and edited all AI-assisted outputs and take full responsibility for the content of the manuscript.

## Authors’ contributions

Juliana Mika Kato: Conceptualization; investigation; formal analysis; writing-original draft.

Maura Salaroli Oliveira: Validation; writing-review and editing.

Luiza Manhezi Shin de Oliveira: Investigation.

Tatiana Tanaka: Validation; investigation.

Gustavo Sakuno: Investigation; formal analysis.

Luciana Santana: Resources; Investigation.

Evangelina da Motta Pacheco Alves de Araujo: Resources.

Joyce Hisae Yamamoto: Supervision; writing-review and editing.

Ruth Miyuki Santo: Supervision; writing-review and editing.

## Funding

This study was approved by the Institutional Research Ethics Committee (CAAE: 67891817.8.0000.0068). A waiver of informed consent was granted due to the retrospective nature of the study and the use of de-identified data, ensuring participant confidentiality.

This study was conducted in accordance with the Strengthening the Reporting of Observational Studies in Epidemiology (STROBE) guidelines.

## Data availability statement

Aggregate data supporting the findings of this study are available within the manuscript and supplementary materials. De-identified individual-level data are available from the corresponding author upon reasonable request, subject to institutional ethical guidelines.

## Declaration of competing interest

The authors declare no conflicts of interest.
